# Hyaluronic acid increases tendon derived cell viability and collagen type I expression in vitro: Comparative study of four different Hyaluronic acid preparations by molecular weight

**DOI:** 10.1186/s12891-015-0735-7

**Published:** 2015-10-06

**Authors:** Leonardo Osti, Martina Berardocco, Viviana di Giacomo, Graziella Di Bernardo, Francesco Oliva, Anna C. Berardi

**Affiliations:** Unit of Arthoscopy and Sports Trauma Surgery, Hesperia Hospital, Modena, Italy; U.O.C. of Immunohaematology and Transfusion Medicine, Laboratory of Stem Cells, Spirito Santo Hospital, via Fonte Romana 8, 65125 Pescara, Italy; Department of Pharmacy, University G. d’Annunzio, Chieti, Italy; U.O.C. of Immunohaematology and Transfusion Medicine, Santo Spirito Hospital, Pescara, Italy; Department of Orthopedics and Traumatology, University of Rome “Tor Vergata” School of Medicine, Rome, Italy

**Keywords:** Hyaluronic acid, Tendinopathy, Human tendon derived cells, Rotator cuff tendons, Shoulder

## Abstract

**Background:**

Hyaluronic Acid (HA) has been already approved by Food and Drug Administration (FDA) for osteoarthritis (OA), while its use in the treatment of tendinopathy is still debated. The aim of this study was to evaluate in human rotator cuff tendon derived cells the effects of four different HA on cell viability, proliferation, apoptosis and the expression of collagen type I and collagen type III.

**Methods:**

An in vitro model was developed on human tendon derived cells from rotator cuff tears to study the effects of four different HA preparations (Ps) (sodium hyaluronate MW: 500-730 KDa - Hyalgan®, 1000 kDa Artrosulfur HA®, 1600 KDa Hyalubrix® and 2200 KDa Synolis-VA®) at various concentrations. Tendon derived cells morphology were evaluated after 0, 7 and 14 d of culture. Viability, proliferation, apoptosis were evaluated after 0, 24 and 48 h of culture. The expression and deposition of collagen type I and collagen type III were evaluated after 1, 7 and 14 d of culture.

**Results:**

All HAPs tested increased viability and proliferation, in dose dependent manner. HAPs already reduce apoptosis at 24 h compared to control cells (without HAPs). Furthermore, HAPs stimulated the synthesis of collagen type I in a dose dependent fashion over 14 d, without increase in collagen type III; moreover, in the presence of Synolis-VA® the expression and deposition of collagen type I was significantly higher as compare with the other HAPs.

**Conclusions:**

HAPs enhanced viability, proliferation and expression of collagen type I in tendon derived cells.

## Background

Non-traumatic rotator cuff tears are the most common shoulder joint disease, and have age-associated incidence, since they are favored by the co-presence of metabolic diseases such as diabetes, thyroid disorders and hypercholesterolemia [1–4]. Conservative treatment of tendinopathies has been increasingly supported by scientific evidence over the last twenty year [5]. Despite decades of study for HA in the conservative treatment of osteoarthritis [6], poor evidence is present in the literature about the indication of this drug for tendinopathies [7]. During tendinopathy and tendon acute rupture has been reported an higher incidence of tenocyte apoptosis and decreased collagen synthesis [8]. Failure of the healing response may occur in genetically-predisposed patients, decreasing the resistance of tendon structures to mechanical load, resulting eventually in tendinopathy, or a tendon tear [4, 9, 10].

Hyaluronic acid (HA) (or “hyaluronan”, or “sodium hyaluronate preparation”) is a high molecular weight glycosaminiglycan consists of the repetition of a disaccharide unit of an N-acetyl-glucosamine and a β-glucuronic acid [11]. Its most *important* physicochemical properties are its capacity to retain water, having a very high hydration ratio, and its visco-elasticity. These two properties are, however, interdependent. Changes in HA concentrations within the extracellular matrix modulate a variety of cellular functions, such as cell migration [12, 13], adhesion [14, 15], and proliferation [16–18]. Several important medical applications of HA have been discovered for joints degeneration [7]. Additionally, high local concentration of HA causes release of endogenous growth factors and stimulates cell–cell interaction, resulting in faster cell proliferation during early stages of in vitro culture. Additional effects reported in clinical animal studies are related to an accelerated healing process in the tendons after repair, and decreased scar formation within the tendons. There has been a lack of specific studies on human shoulder derived cells. Much of the study, has been limited by the lack of the exact phenotype of the tendon derive cells, moreover, the pattern of gene expression is consistent with the presence of mixed population. [19]. Clinical studies in patients with rotator cuff disease ranging from tendinopathy to rotator cuff tears detected a positive influence on the reduction of pain and improved function with no consistent side-effects recorded. Despite the increased awareness of the effective role of HA in regenerative medicine, the therapeutic use of HA for tendinopathies has been poorly studied on human tenocytes in vitro.

In this study, was evaluated the effect of four different HAPs by molecular weight on viability, metabolic activity, apoptosis and collagen type I and collagen type III expression on human rotator cuff tendon tears derived cells.

## Methods

All the procedures described in this investigation were approved by the Ethical Committee of Rome Tor Vergata University. All the patients gave written informed consent to be included in the present study. Tendon samples were harvested from healthy area close to degenerative supraspinatus tendons tear area biopsy specimen in 10 patients were operated arthroscopically for shoulder rotator cuff repair, with a mean age of 63,6 ± 6,9 years. Trauma history, heavy smoking habit or systemic conditions such as thyroid disorders, diabetes, gynecological condition, neoplasia, rheumatic diseases, and any previous or concomitant rotator cuff disease were considered exclusion criteria.

### Tendon cell cultures

Primary human tendon derived cell cultures were established as previously described [20]. In brief, cells were isolated from tissue sample by washing several times with phosphate buffered saline Dulbecco’s W/O Ca and Mg (PBS) + 1 % penicillin/streptomycin (Invitrogen, Life Technologies, Carlsbad, CA, USA). Small pieces of fresh tendon isolated were carefully dissected and mechanically disaggregated with the aid of fine watchmaker forceps to maximize the interface between tissue and medium. Finally, the tendons were immediately placed on Petri dishes of 60 mm in diameter (Greiner CELLSTAR dish, Sigma- Aldrich, Saint Louis, MO, USA), containing 5 mL of α-MEM supplemented with 20 % heat-inactivated foetal calf serum (FCS) and 1 % L-glutamine and 1 % penicillin/streptomycin (Gibco, Invitrogen, Life Technologies) at 37 °C in 5 % CO_2_ and air with a change medium every 2–3 d. Tenocytes were then harvested by StemPro Accutase (Life technologies Carlsbad, CA, USA), and centrifugated at 1,500 rpm for 5 min when the cells migrated out of tendon pieces and reached 60–80 % of confluence (19 day). Collected tendon derived cells were immediately used for culture to avoid phenotype drift with further *in vitro* passages [21]. The phenotype of the tendon derived cells had not demonstrated significant drift as evidence by the gene expression pattern by assessing the expression of gene for scleraxis and genes for collagens α1(I), α2(I) and α1(III) in real-time PCR assays with specific primers (data not shown).

### Tenocyte viability and proliferation

*In vitro* proliferation was determined by the Alamar Blue assay. This test was used to measure the metabolism rate of the cells. The tendon derived cells were seeded with 5×10^3^ vital cells per well in a 96-well plate (Greiner CELLSTAR dish, Sigma-Aldrich), and in triplicates in 100 μl of α-MEM supplemented with 10 % FCS . Cells were cultured as previous described [20]. Briefly, after 24 h, cultured cells were exposed to 4 different hyaluronic acid: Hyalgan MW 500–730 KDa, Artrosulfur HA® MW 1000, Hyalubrix® MW 1600 KDa, Synolis-VA® MW 2200 KDa, their features are shown in Table 1. Three different doses of Hyalgan or Artrosulfur HA® (250 μg/ml, 500 μg/ml and 1000 μg/ml), one doses of Hyalubrix® or Synolis-VA® (1000 μg/ml). HAPs were dissolved in the same culture media used for the entire experiments (α-MEM supplemented with 10 % FCS) and the Ph was adjusted to 7. Untreated cells were used as control. All the cells (HAPs treated and untreated) were cultured in 1 ml of medium. Alamar blue dye test (Serotec, Oxford, UK) was performed to assess cell viability after 0, 24, and 48 h of culture, as previous described [16]. The absorbance was read spectrophotometrically at 570 and 600 nm wavelengths by MicroPlate reader (BioRad, Hercules, CA). The results, obtained as optical density (OD) data, were processed following manufacturer’s instructions and expressed as reduction percentage. The calculation of the of the percentage of alamar blue reduction is as follows according to the manufacture’s protocol:

$$ \frac{\left({\varepsilon}_{\mathrm{ox}}{\lambda}_2\right)\left(\mathrm{A}{\lambda}_1\right) - \left({\varepsilon}_{\mathrm{ox}}{\lambda}_1\right)\left(\mathrm{A}{\lambda}_2\right)\ \mathrm{of}\ \mathrm{test}\ \mathrm{agent}\ \mathrm{dilution}}{\left({\varepsilon}_{\mathrm{red}}{\lambda}_1\right)\ \left(\mathrm{A}'{\lambda}_2\right) - \left({\varepsilon}_{\mathrm{red}}{\lambda}_2\right)\left(\mathrm{A}'{\lambda}_1\right)\ \mathrm{of}\ \mathrm{untreated}\ \mathrm{positive}\ \mathrm{growth}\ \mathrm{control}}\kern0.5em \times 100 $$

**Table 1:** Features of Hyaluronic Acids preparations tested

Commecial Name	Hyalgan®	Artrosulfur HA®	Hyalubrix®	Synolis-VA®
Active Substance	Linear Sodium Hyaluronate	Linear Sodium Hyaluronate	Linear Sodium Hyaluronate	Linear Sodium Hyaluronate + Sorbitol (4 %) (limits the HA degradation)
Molecular Weight	600–730 KDa	1000 KDa	1600 KDa	2000 KDa
Source	Rooster Combs	Bacterial Fermentation	Bacterial Fermentation	Bacterial Fermentation
Doses Tested	250 μg/ml	250 μg/ml	1000 μg/ml	1000 μg/ml
	500 μg/ml	500 μg/ml		
	1000 μg/ml	1000 μg/ml		
Manufacturer	Fidia Farmaceutici s.p.a., Abano Terme (PD), Italy	Laborests.p.a., Nerviano (MI), Italy	Fidia Farmaceutici s.p.a. Abano Terme (PD), Italy	Anteis s.a., Geneva, Switzerland

In the formula ελ_1_ and ελ_2_ are constant representing the molar extinction coefficient of alamar blue at 540 nm and 630 nm, respectively, in the oxidized (ε_ox_) and the reduced (ε_red_) forms. Aλ_1_ and Aλ_2_ represent absorbance of test wells at 540 nm and 630 nm, respectively. A’λ_1_ and A’λ_2_ represent absorbance of negative control wells at 540 and 630 nm, respectively. The values of % alamar blue reduction were corrected for background values of negative controls containing medium without cells.

Finally, in parallel trypan blue exclusion assay was performed. The tendon derived cells were seeded with 10^4^ vital cells per well in a 24-well plate (Greiner CELLSTAR dish, Sigma-Aldrich), and in triplicates in 1 ml of α-MEM supplemented with 10 % FCS. After 0, 24 and 48 h the cultures were detached, collected and counted (Nikon Instruments INC., Melville, NY, USA) in the Burker chamber with vital dye Trypan Blue (Stem Cells Technologies, Vancouver, Canada) to evaluate cell viability.

### Apoptosis induction

Hydrogen peroxide (H_2_O_2_) was used as an inducer of apoptosis as previously described [22]. The tendon derived cells were seeded with 10^5^ vital cells per well in a 6-well plate in 4 ml of α-MEM supplemented with 10 % FCS. After 24 h, the medium was removed, and the cultured cells were treated with H_2_O_2_ (2 mM) in α-MEM and 10 % FCS with or without Hyalgan®, Artrosulfur HA®, Hyalubrix® and Synolis-VA® (1000 μg/ml) for a further 24 h. A negative control was prepared by incubating cells in the absence of both inducing agent and HAPs. The PE Annexin V/Dead Cell Apoptosis Kit with SYTOX® Green for Flow Cytometry (Invitrogen, Life Technologies) was used to detect apoptosis by flow cytometry, cells were harvested, and processed according to the manufacturer's instruction. This product detects the externalization of phosphatidylserine in apoptotic cells using recombinant annexin V conjugated to the orange fluorescent phycobiliprotein R-PE, and dead cells using SYTOX® Green nucleic acid stain. After treatment with both probes, apoptotic cells show orange fluorescence, dead cells show green fluorescence, and live cells show little or no fluorescence. Fluorescence-activated cell sorting analysis was carried out using a FC500 flow cytometer (FL1 and FL3 detector in a log mode) using the CXP analysis software (Beckmann Coulter, FL, USA).

### Immunofluorescence staining

The tendon derived cells were seeded with 5×10^3^ vital cells per well in a 2-well chamber slides (Thermo Fisher Scientific*, Inc.,* Rochester, NY, USA), in triplicates and cultured as previous described [23]. After 1, 7 and 14 d of culture the tendon derived cells were fixed with pure acetone for 10 min at −20 °C. Then, washed a few minutes with PBS. Cells were incubated for 30 min at room temperature with PBS containing 5 % of Bovine Serum Albumin (BSA) (Kedrion Group S.P.A., Lucca, Italy) for protein blockage. Primary antibodies for Anti-type I (1:2000), Anti-type III collagen molecules (1:500) (Sigma-Aldrich), and secondary antibodies fluorochrome were diluted in PBS containing 5 % BSA. Cells were incubated overnight at 4 °C with primary antibodies, 1 h with the appropriate secondary antibody fluorochrome at room temperature and then washed a few times with PBS containing 5 % BSA. Molecule’s staining Alexa Fluor 488 (Life Tecnologies) was used for type I collagen and Alexa Fluor 568 (Life Tecnologies) for type III collagen. After washing with PBS plus 5 % BSA, slides were mounted with 25 μL VECTASHIELD® Hard Set Mounting Medium and then were examinated with ECLIPSE Ti-U inverted, fluorescent microscope (Nikon Instruments INC., Melville, NY, USA). For image analysis all digital images were captured with NIS-Elements Imaging Software (Nikon Instruments INC.). As previously described [23, 24], slides were examined independently by two experienced operator and one researcher, with a double-blind method. The total fluorescence intensity of the area ≥ 10 frames from each slides was determined. The intensity level was normalized with the control cells untreated. Fully automated image analysis improve the accuracy of detection and categorization of collagen staining, making this technique more sensitive, specific and thus suitable for use in quality assurance results.

### Statistical analysis

Data are typical results from a minimum of three replicated independent experiments and are expressed as mean ± SD. Comparison of individual treatment was conducted using Student’s *t* test. Statistical significance in comparison with the corresponding control values was indicated by *P < 0.05 versus control.

## Results

### Tendon derived cells viability

**Fig. 1:**
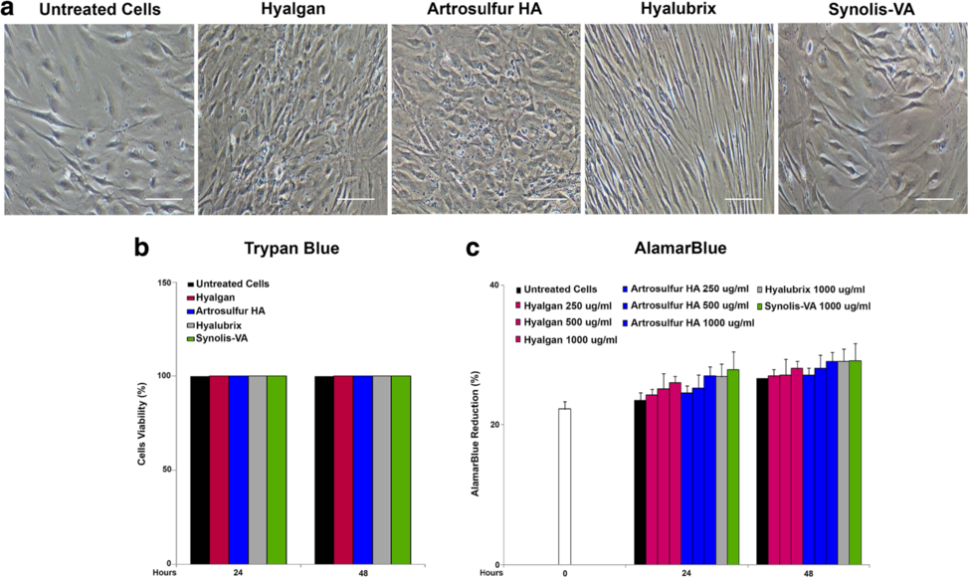
Effects of Hyalgan®, Artrosulfur HA®, Hyalubrix® and Synolis-VA® on morphologic change cell viability and cell metabolic activity. Tendon derived cells were treated with different concentrations of Hyalgan, Artrosulfur HA®, Hyalubrix® and Synolis-VA® for different time periods. **a**. after treatment for 14 d the morphology of the cells treated with all the HAPs was photographed (1000ug/ml). Magnification, ×100. **b**. Absolute number of live cells was calculated using trypan blue exclusion. **c**. Cell metabolic activity was evaluated with the Alamar Blue method. All tests and determinations were repeated in triplicate. The metabolic activity rate was calculated by subtracting the background OD value (complete culture medium without cells) from the OD value from each test well (see materials and methods section). Hyalgan®, Artrosulfur HA®, Hyalubrix®, Synolis-VA® increased cell activity in a concentration-dependent pattern and with regard to time. No statistically significant difference were observed from control according to Student's *t* test

Tendon derived cells morphology was evaluated under a light microscope at 0, 7 and 14 d. The cells maintained their normal, bipolar, spindle shape and cell processes, during the whole study period for each of the sets of culture conditions; cellular morphology remained unaltered for up to 14 d in all the experimental groups (Fig. 1a). Results from the trypan blue exclusion assay showed that none of the HAPs reduced cell viability (Fig. 1b). After 48 h of exposure, living cells are in similar numbers when exposed to HAPs compared to the control. Metabolic tests provide some information concerning the activity of cells (Fig. 1c). Alamar blue confirmed an increase in the metabolic activity of tendon derived cells for all the HAPs utilized, when compared to untreated cells (Fig. 1c). In particular, all HAPs induced cell-activity most effectively at 1000 μg/ml (Fig. 1c, Table 2). The highest increase was obtained at 48 h for all the HA treatments. However, as reported in Fig. 1c, there are no significant statistical differences between all the various HAPs.

**Table 2:** Summary of results

METABOLIC ACTIVITY			
Cells	0 h	24 h	48 h
Untreated	22,25 % ± 1,1 %	23,18 % ± 1,3 %	26,69 % ± 0,1 %
Hyalgan® 250 μg/ml		23,68 % ± 0,9 %	26,92 % ± 1,4 %
Hyalgan® 500 μg/ml		25,37 % ± 2,2 %	27,14 % ± 1,9 %
Hyalgan® 1000 μg/ml		25,54 % ± 1,2 %	27,71 % ± 1,3 %
Artrosulfur HA ® 250 μg/ml		24,13 % ± 1,1 %	27,16 % ± 1 %
Artrosulfur HA ® 500 μg/ml		25,47 % ± 1,8 %	27,7 % ± 1,7 %
Artrosulfur HA ® 1000 μg/ml		26,9 % ± 1,5 %	28,55 % ± 1,4 %
Hyalubrix® 1000 μg/ml		26,85 % ± 1,9 %	28,56 % ± 1,9 %
Sinolis -VA® 1000 μg/ml		27,44 % ± 2,2 %	28,58 % ± 2,1 %
APOPTOSIS			
Cells	Live cells	Early	Late
Proliferant control	91,2 % ± 0,2 %	1,8 % ± 0,2 %	4 % ± 0,8 %
Apoptotic control	20,5 % ± 0,7 %	10,9 % ± 0,1 %	66,8 % ± 0,1 %
Hyalgan® 1000 μg/ml	31,2 % ± 9,4 %	12,4 % ± 2,5 %	54,4 % ± 7,1 %
Artrosulfur HA ® 1000 μg/ml	20,7 % ± 8 %	9,2 % ± 0,2 %	66,8 % ± 0,7 %
Hyalubrix® 1000 μg/ml	34,9 % ± 6,7 %	11,2 % ± 1,3 %	52,4 % ± 5,3 %
Sinolis -VA® 1000 μg/ml	31,3 % ± 3,5 %	12 % ± 1,7 %	55 % ± 1,9 %
MEAN GREEN INTENSITY			
Cells	Day 0	Day 7	Day 14
Untreated	0,5	4,2 ± 0,9	8,6 ± 1
Hyalgan® 1000 μg/ml		6,7 ± 1,1	12,3 ± 1,3
Artrosulfur HA ® 1000 μg/ml		6,4 ± 1,5	12,4 ± 1,3
Hyalubrix® 1000 μg/ml		8,9 ± 2,2	10,9 ± 2,1
Sinolis -VA® 1000 μg/ml		8,6 ± 2,5	16,1 ± 3

### Apoptosis induction

**Fig. 2:**
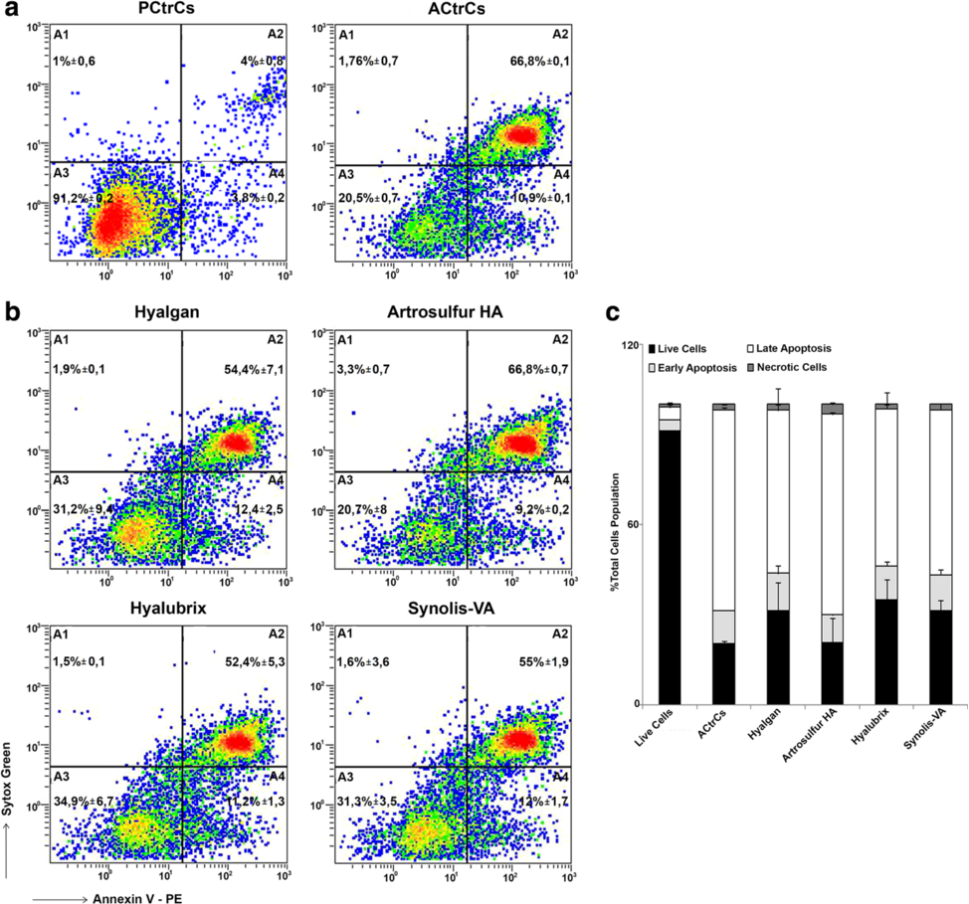
The apoptosis rate in tendon derived cells. **a**. The apoptosis was induced with H2O2 (apoptotic control cells [ACtrCs]) and treated with Hyalgan®, Artrosulfur HA®, Hyalubrix® and Synolis-VA® (1000 ug/ml). Apoptosis rate of cells in each sample were detected in a population of 10,000 cells analyzed by flow cytometry. **b**. The percentage of apoptotic cells in Hyalgan®, Artrosulfur HA®, Hyalubrix® and Synolis-VA® decreased compared to the proliferate control cells (PCtrCs) (without HA). Flow charts: (A_1_), upper left quadrant annexin V-negative and sytox green-positive cells indicate necrotic cells; (A_2_), upper right quadrant, annexin V and sytox green -positive cells represent late apoptotic cells. (A_3_), lower left quadrant annexin V-negative and sytox green-negative cells indicate live cells; (A_4_), lower right quadrant, annexin V positive and sytox green-negative cells represent early apoptotic cells. Both early and late apoptotic cells were calculated as the incidence of apoptotic cell. The numbers indicate percentage of total gated cells (mean ± S.D.; n = 3). **c**. % of cells population of live cells, early apoptotic cells, late apoptotic cells and necrotic cells. Graph showing the average % of results obtained in 3 independent experiment. No significant differences were detected (mean ± S.D.; *n* = 3)

To verify whether or not they counteracted apoptosis in tendon derived cells, the Annexin V experiment was performed. Cells were plated and H_2_O_2_ induction was performed for 24 h to induce apoptosis. Concurrently, tendon derived cells were separately exposed (or not, for the untreated sample) to Hyalgan®, Artrosulfur HA®, Hyalubrix®, and Synolis-VA® (1000 μg/ml). Staining cells simultaneously with PE-Annexin V (red fluorescence) and the non-vital dye, Sytox Green (green fluorescence), allowed, using bivariate analysis, discrimination between intact cells (Annexix V^−^Sytox Green^−^), early apoptotic (Annexix V^+^Sytox Green^−^) and late apoptotic (Annexix V^+^Sytox Green^+^) and necrotic cells (Annexix V^−^Sytox Green^+^) (Fig. 2a). The treatment of tendon derived cells with Hyalgan®, Artrosulfur HA®, Hyalubrix®, or Synolis-VA® (all at 1000 μg/ml) caused a sizable decrease in apoptosis, as clearly shown in Fig. 2b. The percentage of vital cells, at 24 h following Hyalgan®, Artrosulfur HA®, Hyalubrix®, or Synolis-VA® exposure, increased compared to the control (33.14, 24.01, 36.31, 33.04 and 22.25 % respectively) (Annexix V^−^, Sytox green^−^; bottom left quadrant) (Fig. 2c, Table 2).

### Immunofluorescence staining

**Fig. 3:**
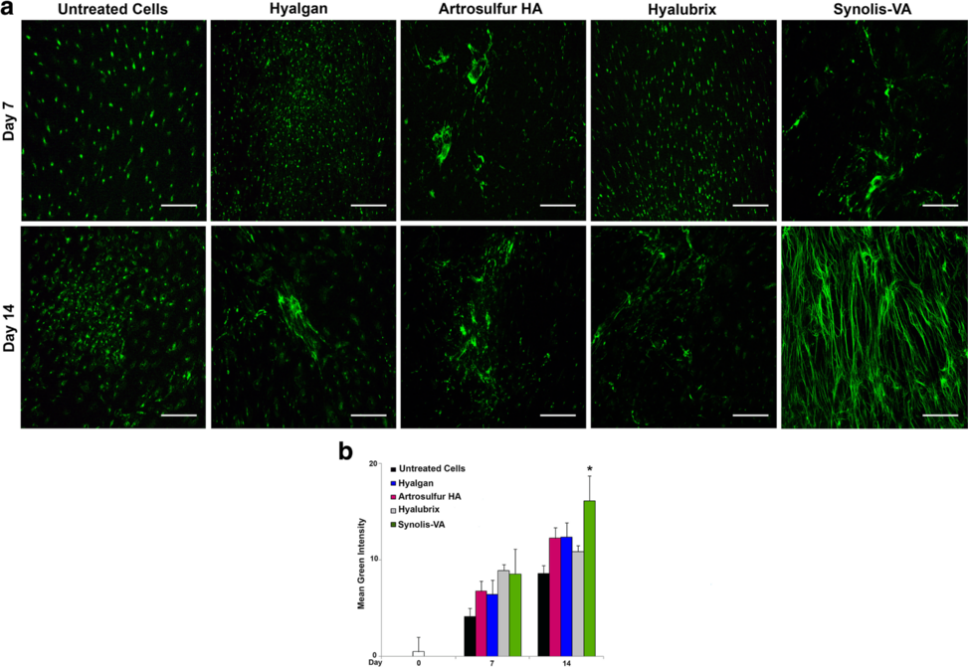
Collagen type I, expression of tendon derived cells in vitro culture isolated from 9 healthy patients, and staining as described in material and methods, section Immunofluorescence Staining. Representative images from 4 independent experiments. **a**. Expression of collagen type I after 7 and 14 d of culture in green fluorescence and in regular light image showing the tendon derived cells. **b**. Quantization of the immunohistochemistry in a. Synolis-VA® induced the expression of collagen type I significantly after 14 d of in vitro culture. The mean fluorescent intensity/pixel was measured and expressed to the corresponding tendon derived cells. Collagen type I Intensity (Total Area was quantified by anti-collagen type I) it was measured by Nikon software. Data are expressed as mean ± SD for 4 independent experiments for samples run in triplicate. Scale bar (a.): 50 μm

Next, we determined and measured the type of collagen deposited by tendon derived cells after stimulation with Hyalgan®, Artrosulfur HA®, Hyalubrix®, or Synolis-VA®. Collagen accumulation was evaluated by immunofluorescent staining of cells cultured on chamber slides. Furthermore, the expression of collagen type I was higher in Synolis-VA® than in the presence of Hyalgan®, Artrosulfur HA®, or Hyalubrix®, and was significantly higher compared to untreated cells used as a control (Fig. 2a, b). In detail, immunofluorescent staining at day 7 only revealed production of collagen type I from the tendon derived cells in intracytoplasmic staining (Fig. 3a). Moreover, at day 14 expression and production of Collagen type I had increased in the Hyalgan®, Artrosulfur HA®, Hyalubrix® and Synolis-VA® (Fig. 3a, b). Moreover, Synolis-VA® induced the most significant expression of collagen type I after 14 d (Fig. 3, Table 2). Collagen type III was not found to be present in any culture conditions (data not shown).

## Discussion

These results suggest a binary role for HA on tendon cells - directly, on tendon derived cells metabolic activity, and on tenocyte Collagen type I production. It was highlighted that Hyalgan, Artrosulfur HA®, Hyalubrix® and Synolis-VA® regulate cell activity of tendon derived cells. All HAPs increased cell-metabolic activity and most effectively at 1000 μg/ml and at 48 h (Fig. 1c). HA modulate a number of biological process including cell apoptosis. The results show a decrease rate of the apoptosis when the tendon derived cells were exposed to the HAPs. Collagen metabolism has been reported to be affected by HA [25, 26]. HA stimulated the synthesis of collagen type I, in a dose dependent manner over 14 d. This increase in expression leads to collagen synthesis and accumulation. It should be noted that Synolis-VA® induced the most significant expression of collagen type I at 14d of culture. In contrast, no HAPs induced any expression of collagen type III, which is normally less abundant in tendons, and only increases in concentration during the early phase of remodeling [27] and in tendinopathy [20, 28]. The lack of collagen type III production under HA stress should be considered a protective factor for tendons. The results obtained are consistent with a previous study of Yamada and coworkers, even if a different methods focused directly on the collagen type I and III proteins was used [29].

Considering the results of this study, the three different molecular weight of HAPs tested seems not exert any effects on tendon derived cells in vitro, while is clear the essential importance of the concentrations and of the timing of exposure. The most significant expression of collagen type I at 14 d of culture of Synolis-VA® can be explained to the presence of Sorbitol (4 %) that limits the HA degradation, allowing an higher local concentration of the drug. Translating these considerations in the clinical practice, HA are effectives on human tenocytes and extracellular matrix of rotator cuff, with no essential differences among HA available in the market. There are many biological questions that remain to be answered, and translational factors to resolve. Although, this *in vitro* model shows some role played from HA on tendon derived cells, the study have some limitations. First of all, probably these results cannot be generalized for other tendon derived cells from other sources; furthermore *in vitro* environment, rich of nutrients and oxygen is very different from the diseased environment. The complexity of the extracellular matrix of tendons and its relationship with tenocytes during physiological homeostasis, disease and healing process, attest that is reductive to investigate only the effect of HAPs on collagen type I and III. As soon as possible there is need to widen the knowledge of the effects of HAPs on the main proteins of the extracellular matrix of tendons.

Despite we tested the three different molecular weight HA, perhaps more HAPs and different concentration need to be tested in the same way we did to confirm which should have the best *in vitro* results.

Obviously, it is advocate randomized control studies on the use of HA in the conservative treatment of tendinopathy and in selected patients with rotator cuff tears, in order to understand and clarify best timing, doses, intervals of injections and, finally, full clinical confirmation of effectiveness.

## Conclusion

In conclusion, HAPs in dose dependent manner but not related to the molecular weight, induces increase of cells activities, decrease of apoptosis of tendon derived cells Collagen type I protein secretion. Taken together, these results strengthen a physiological role of HA in the homeostasis of tendons and has implications for regenerative medicine.

## References

[1] Oliva F, Osti L, Padulo J, Maffulli N (2014). Epidemiology of the rotator cuff tears: a new incidence related to thyroid disease. Muscles Ligaments Tendons J.

[2] Osti L, Rizzello G, Panascì M, Denaro V, Maffulli N (2011). Full thickness tears: retaining the cuff. Sports Med Arthosc.

[3] Oliva F, Berardi AC, Misiti S, Maffulli N (2013). Thyroid hormones and tendon: current views and future perspectives. Concise review. Muscles Ligaments Tendons J.

[4] Klatte-Schulz F, Pauly S, Scheibel M, Greiner S, Gerhardt C, Schmidmaier G (2012). Influence of age on the cell biological characteristics and the stimulation potential of male human tenocyte-like cells. Eur Cell Mater.

[5] Loppini M, Maffulli N (2012). Conservative management of tendinopathy: an evidence-based approach. Muscles Ligaments Tendons J.

[6] Rutjes AW, Jüni P, da Costa BR, Trelle S, Nüesch E, Reichenbach S (2012). Viscosupplementation for osteoarthitis of the knee: a systematic review and meta-analysis. Ann Intern Med.

[7] Abate M, Schiavone C, Salini V (2014). The use of hyaluronic acid after tendon surgery and in tendinopathies. Biomed Res Int.

[8] Giai Via A, De Cupis M, Spoliti M, Oliva F (2014). Clinical and biological aspects of rotator cuff tears. Muscles Ligaments Tendons J.

[9] Yuan J, Wang MX, Murrell GA (2003). Cell death and tendinopathy. Clin Sports Med.

[10] Benson RT, McDonnell SM, Knowles HJ, Rees JL, Carr AJ, Hulley PA (2010). Tendinopathy and tears of the rotator cuff are associated with hypoxia and apoptosis. J Bone Joint Surg Br.

[11] Meyer K (1958). Chemical structure of hyaluronic acid. Fed Proc.

[12] Melrose J, Numata Y, Ghosh P (1996). Biotinylated hyaluronan: a versatile and highly sensitive probe capable of detecting nanogram levels of hyaluronan binding proteins (hyaladherins) on electroblots by a novel affinity detection procedure. Electrophoresis.

[13] Chen WY, Grant ME, Schor AM, Schor SL (1989). Differences between adult and fetal fibroblasts in the regulation of hyaluronate synthesis: correlation with migratory activity. J Cell Sci.

[14] Klein ES, Asculai SS, Ben-Ari GY (1996). Effects of hyaluronic acid on fibroblast behavior in peritoneal injury. J Surg Res.

[15] Hall CL, Wang C, Lange LA, Turley EA (1994). Hyaluronan and the hyaluronan receptor RHAMM promote focal adhesion turnover and transient tyrosine kinase activity. J Cell Biol.

[16] Wiig M, Abrahamsson SO, Lundborg G. Effects of hyaluronan on cell proliferation and collagen synthesis: a study of rabbit flexor tendons *in vitro*. J Hand Surg Am. 1996;21(4):599–604. 10.1016/S0363-5023(96)80010-4 8842950

[17] Bernard E, Hornebeck W, Robert L (1994). Effect of hyaluronan on the elastase-type activity of human skin fibroblasts. Cell Biol Int.

[18] Matuoka K, Namba M, Mitsui Y (1987). Hyaluronate synthetase inhibition by normal and transformed human fibroblasts during growth reduction. J Cell Biol.

[19] Dean BJ, Snelling SJ, Dakin SG, Murphy RJ, Javaid MK, Carr AJ (2015). Differences in glutamate receptors and inflammatory cell numbers are associated with the resolution of pain in human rotator cuff tendinopathy. Arthritis Res Ther.

[20] Oliva F, Berardi AC, Misiti S, Verga Falzacappa C, Iacone A, Maffulli N (2013). Thyroid hormones enhance growth and counteract apoptosis in human tenocytes isolated from rotator cuff tendons. Cell Death Disease.

[21] Yao L, Bestwick CS, Bestwick LA, Maffulli N, Aspden RM (2006). Phenotypic drift in human tenocyte culture. Tissue Eng.

[22] Yuan J, Murrell GA, Trickett A, Wang MX (2003). Involvement of cytochome c release and caspase-3 activation in the oxidative stress-induced apoptosis in human tendon fibroblasts. Biochimica et Biophysica Acta.

[23] Berardi AC, Oliva F, Berardocco M, la Rovere M, Accorsi P, Maffulli N (2014). Thyroid hormones increase collagen type Iand cartilage oligomeric matrix protein (COMP) expression in vitro human tenocytes. Muscles Ligaments Tendons J.

[24] Hanna S, Khalil B, Nasrallah A, Saykali BA, Sobh R, Nasser S (2014). StarD13 is a tumor suppressor in breast cancer that regulates cell motility and invasion. Int J Oncol.

[25] Evanko SP, Tammi MI, Tammi RH, Wight TN (2007). Hyaluronan-dependent pericellular matrix. Adv Drug Deliv Rev.

[26] Karna E, Miltyk W, Surazyński A, Pałka JA (2008). Protective effect of hyaluronic acid on interleukin-1-induced deregulation of beta1-integrin and insulin-like growth factor-I receptor signaling and collagen biosynthesis in cultured human chondrocytes. Mol Cell Biochem.

[27] Oliva F, Gatti S, Porcellini G, Forsyth NR, Maffulli N (2012). Growth factors and tendon healing. Med Sport Sci..

[28] Maffulli N, Ewen SW, Waterston SW, Reaper J, Barrass V (2000). Tenocytes from ruptured and tendinopathic achilles tendons produce greater quantities of type III collagen than tenocytes from normal achilles tendons. An in vitro model of human tendon healing. Am J Sports Med.

[29] Yamada T, Gotoh M, Nakama K, Mitsui Y, Higuchi F, Nagata K (2007). Effects of hyaluronan on cell proliferation and mRNA expression of procollagens alpha 1 (I) and alpha 1 (III) in tendon-derived fibroblasts from patients with rotator cuff disease: an in vitro study. Am J Sports Med.

