# Erratum to: Hyaluronic acid increases tendon derived cell viability and collagen type I expression in vitro: Comparative study of four different Hyaluronic acid preparations by molecular weight

**DOI:** 10.1186/s12891-015-0785-x

**Published:** 2015-11-04

**Authors:** Leonardo Osti, Martina Berardocco, Viviana di Giacomo, Graziella Di Bernardo, Francesco Oliva, Anna C. Berardi

**Affiliations:** Unit of Arthoscopy and Sports Trauma Surgery, Hesperia Hospital, Modena, Italy; U.O.C. of Immunohaematology and Transfusion Medicine, Laboratory of Stem Cells, Spirito Santo Hospital, via Fonte Romana 8, Pescara, 65125 Italy; Department of Pharmacy, University G. d’Annunzio, Chieti, Italy; U.O.C. of Immunohaematology and Transfusion Medicine, Santo Spirito Hospital, Pescara, Italy; Department of Orthopedics and Traumatology, University of Rome “Tor Vergata” School of Medicine, Rome, Italy

## Erratum

After publication of the article [1], one of the corresponding authors noticed that there was information mistakenly omitted from the ‘Acknowledgements’ section. “Blood Donors Association FIDAS Pescara” should have been acknowledged. The section should have read as follows, which has been updated on the original article:
